# Eruptive gouty panniculitis associated with idiopathic multicentric Castleman disease (iMCD-NOS)

**DOI:** 10.1016/j.jdcr.2026.07.047

**Published:** 2026-07-31

**Authors:** Max Wu, Karen Koch, Doug White, Duncan Lamont

**Affiliations:** a The University of Sydney, Faculty of Medicine, Royal Prince Alfred Hospital, Sydney, Australia; b Dermatologist, Te Whatu Ora Health Waikato (Waikato Hospital), Honorary Senior Clinical Lecturer, Waikato School of Medicine (Auckland University), Hamilton, New Zealand; c Rheumatologist, Te Whatu Ora Health Waikato (Waikato Hospital), Honorary Senior Clinical Lecturer, Waikato School of Medicine (Auckland University), Hamilton, New Zealand; d Honorary Professor Waikato University Medical School, Hamilton, New Zealand; e Pathologist, Te Whatu Ora Health Waikato (Waikato Hospital), Hamilton, New Zealand

**Keywords:** Castleman disease, gout, iMCD-NOS, panniculitis

## Introduction

Gouty panniculitis is a rare form of panniculitis where monosodium-urate crystals are deposited in subcutaneous adipose tissue. Castleman disease encompasses a heterogeneous group of clinicopathologic disorders characterized by a spectrum of hematologic, immunologic, and systemic manifestations.^1^ Multiple subtypes exist including idiopathic multicentric Castleman disease not otherwise specified (iMCD-NOS). This is a rare, life-threatening variant accounting for approximately 33% to 58% of all MCD cases.^1^

We present a 42-year-old Pacific Islander man in whom eruptive gouty panniculitis coincided with the onset of iMCD-NOS, confirmed on lymph-node biopsy, with good response to siltuximab. This case highlights the importance of considering an underlying lymphoproliferative disease in the setting of gouty panniculitis. The exact mechanism remains uncertain.

## Case presentation

A 42-year-old male of Pacific-Islander descent with a background of type 2 diabetes mellitus (T2DM) and stage 3B chronic kidney disease (CKD) presented with a 2-month history of rapidly developing, firm, erythematous to violaceous, calcified subcutaneous nodules on the lower limbs, which progressively enlarged, ulcerated, and discharged serosanguinous pus before developing secondary scale and crusting. This acute cutaneous presentation occurred against a background of long-standing polyarticular tophaceous gout affecting the flexor and extensor surfaces of both lower limbs and both first metatarsal-phalangeal joints (Figs 1, *A* and *B*). Concomitantly, he developed new-onset generalised pruritus with linear excoriations over the abdomen, forearms, and back (Fig 1, *C*).

**Fig 1:**
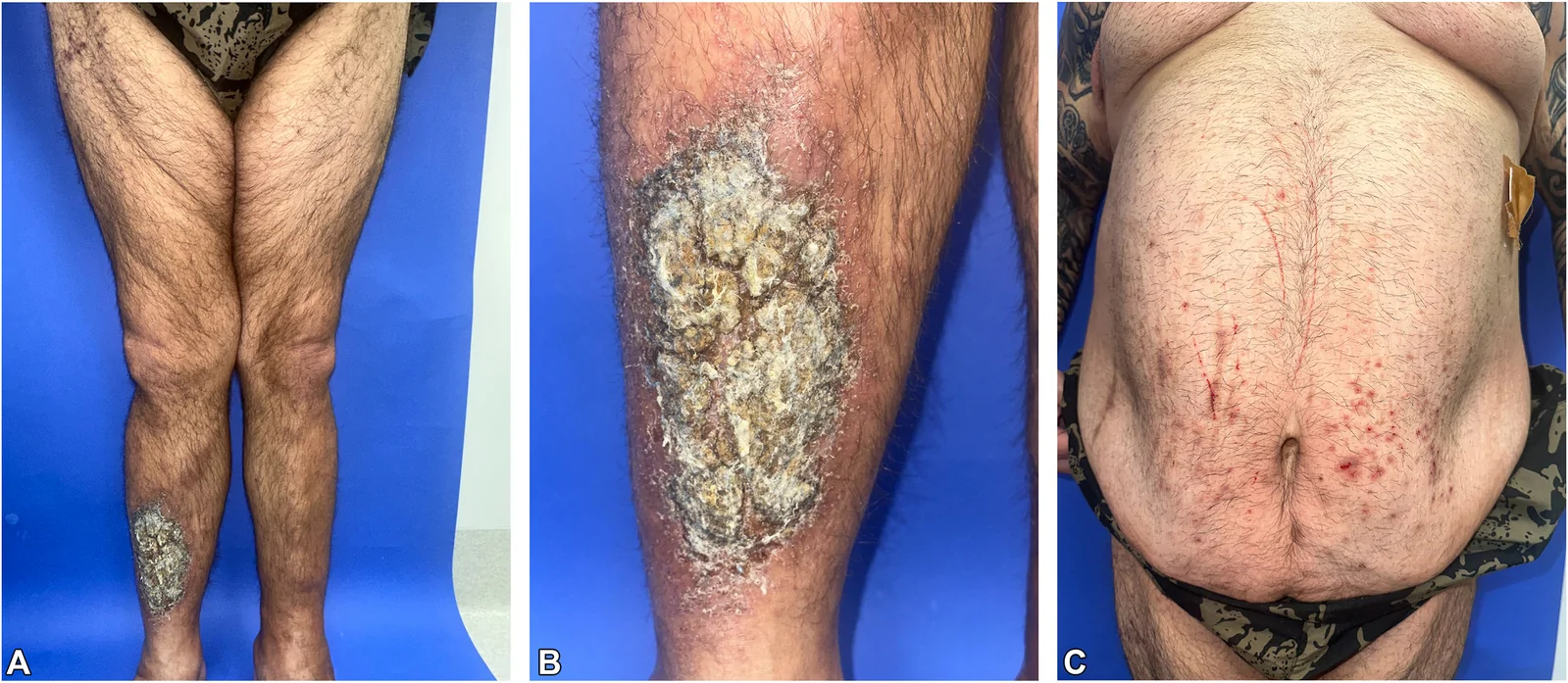
Clinical manifestations of eruptive gouty panniculitis and iMCD-NOS. **A,** Bilateral lower extremity overview demonstrating a hyperkeratotic, scaly plaque on the right anterior shin, which initially presented as a nodular subcutaneous lump. **B,** Close-up view of the right anterior shin showing an extensive hyperkeratotic, scaly plaque with overlying crusted exudate, which enlarged progressively and discharged pus. **C,** Generalized pruritus with linear excoriations and eczematous plaques over the abdomen and torso, consistent with cutaneous manifestations of iMCD-NOS.

His T2DM was well-controlled, and CKD was stable (baseline creatinine 130-160 μmol/L). His polyarticular tophaceous gout had been diagnosed many years prior; notably, serum uric acid levels had consistently fallen within the normal-range,^2^ a recognized phenomenon in established tophaceous disease, particularly in patients on urate-lowering therapy.^3^ His gout was stable and managed with febuxostat, initiated 2 years prior to his acute symptoms. There were no changes to his medications in the preceding 12 months. None of his chronic medications are known to aggravate gout.

He had systemic symptoms beginning 18-months prior to his cutaneous presentation with fatigue, malaise, night sweats, and significant weight loss. Initial CT imaging demonstrated diffuse lymphadenopathy involving the axillary, abdominal, and inguinal regions. Blood tests demonstrated normochromic normocytic anemia, markedly elevated CRP (140 mg/L) and ESR, normal LDH and polyclonal hypergammaglobulinaemia (total IgG 4060 mg/dL). Workup for HHV-8, HIV, EBV, CMV, tuberculosis, syphilis, Hepatitis B/C and IgG4 disease was negative, and there was no evidence of lymphoma, plasma-cell neoplasm, or autoimmune connective-tissue disease (RF, ANA, C3, C4). His serum urate was 0.21 mmol/L and remained within the normal range throughout.

An incisional biopsy from the right posterior calf showed calcified gouty deposits with characteristic basophilic, crystalline amorphous deposits surrounded by extensive granulomatous inflammation extending from the deep dermis into subcutaneous fat. The inflammatory infiltrate comprised predominantly lymphocytes and neutrophils, alongside numerous foam cells and multinucleated giant cells, with fibrosis within the fat lobules consistent with lobular panniculitis (Fig 2, *A*-*C*). Radiological imaging confirmed extensive calcium deposits throughout the dermis and subcutis (Fig 3, *A* and *B*).

**Fig 2:**
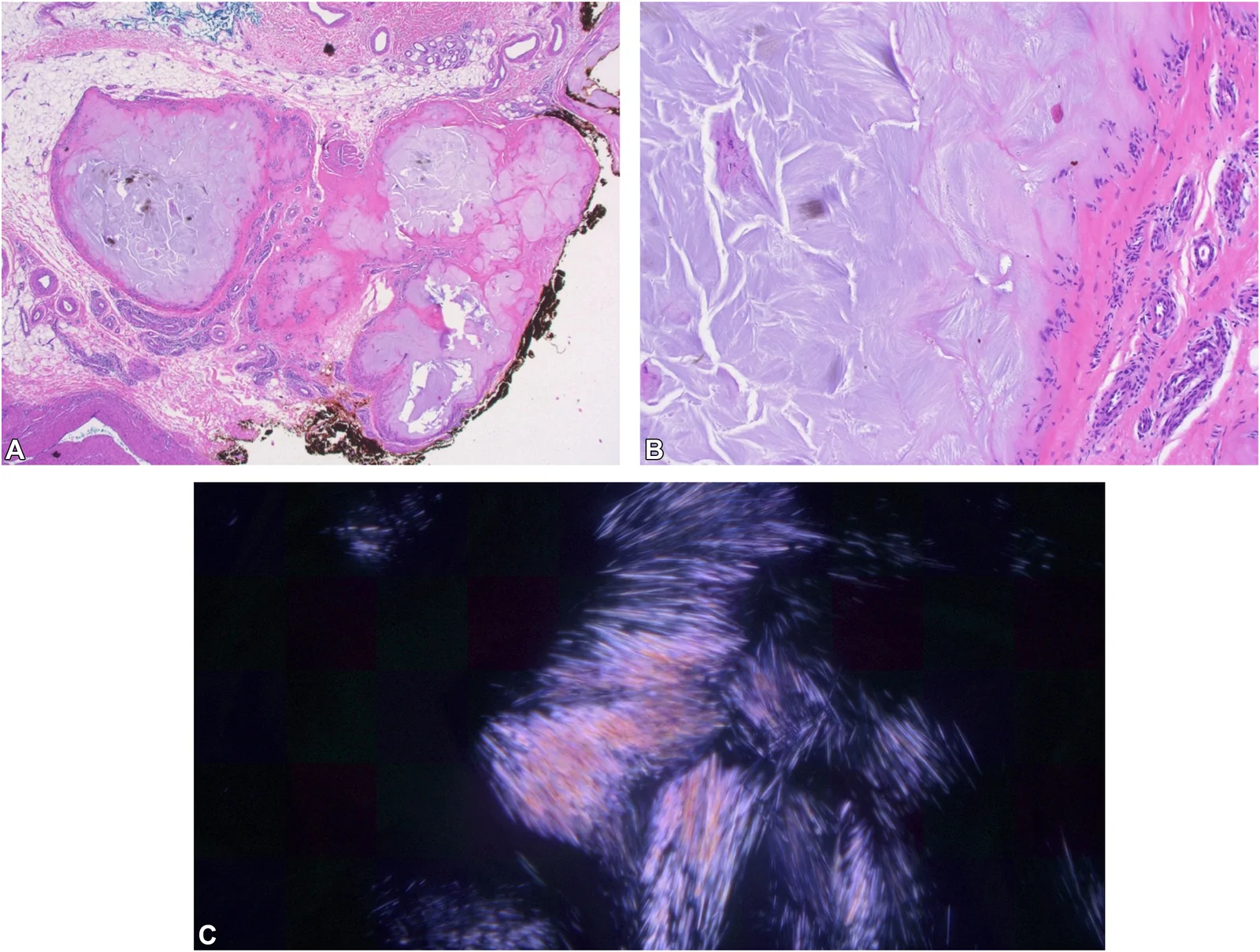
Histopathologic findings of eruptive gouty panniculitis. **A,** Low-power view (×20 magnification) of the deep dermis and subcutaneous fat demonstrating large, basophilic, crystalline amorphous deposits consistent with tophaceous gout. **B,** High-power view (×200 magnification) of the same region showing the characteristic feathery appearance of monosodium-urate crystals with surrounding granulomatous inflammation. **C,** Polarized light microscopy (×400 magnification) revealing needle-shaped, negatively birefringent crystals characteristic of a gouty tophus.

**Fig 3:**
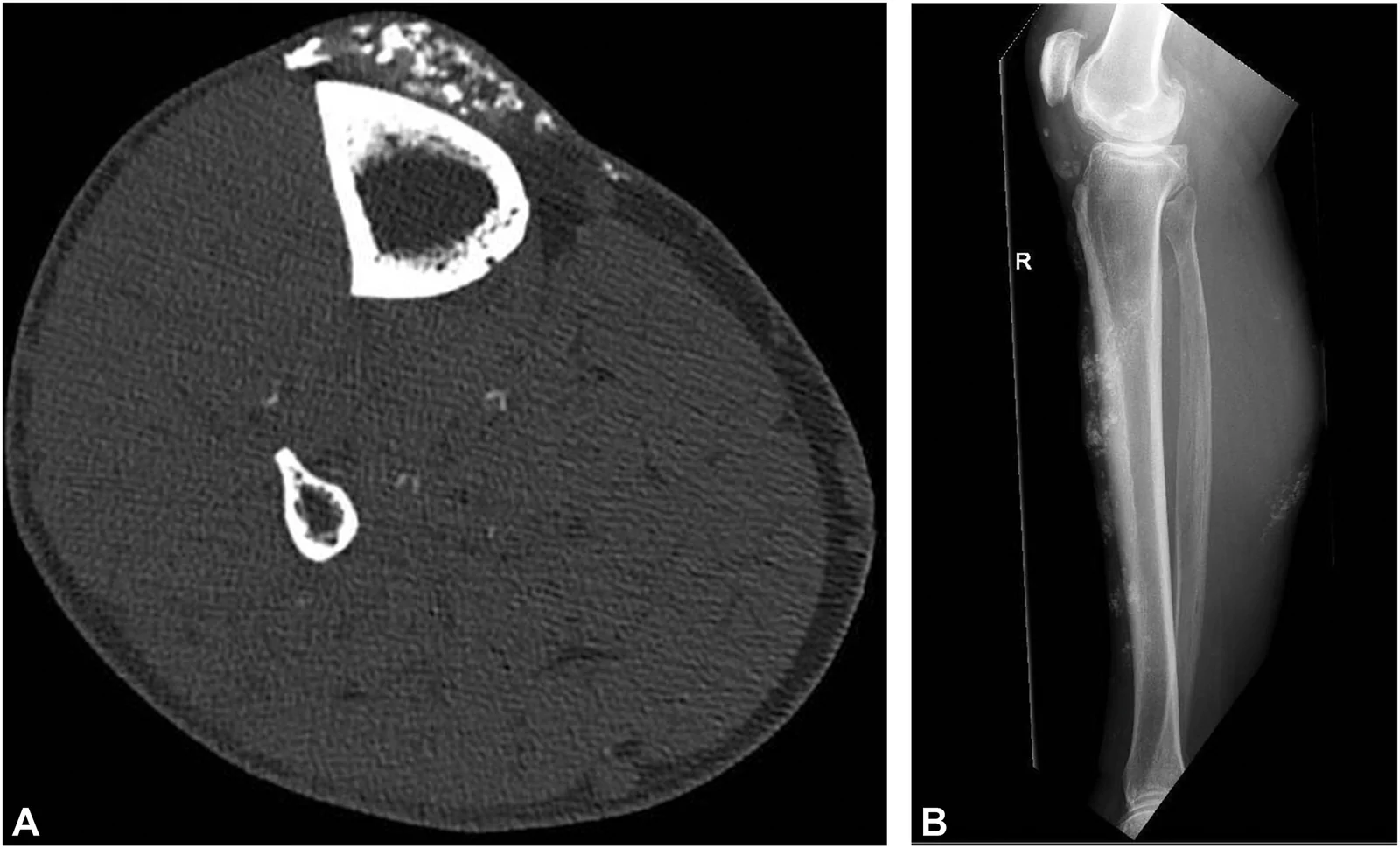
Radiological imaging of subcutaneous calcium deposits: **(A)** Axial CT scan of the lower leg demonstrating multiple hyperdense calcific deposits within the dermis and subcutaneous tissues. **B,** Lateral plain radiograph of the lower leg showing extensive soft tissue radio-opacities corresponding to the sites of eruptive gouty panniculitis.

Lymph-node core biopsy (left axillary node, 8-months from symptom onset) demonstrated a polymorphous lymphoid infiltrate with small B-cell follicles and a prominent interfollicular polyclonal plasma cell proliferation (grade 3 plasmacytosis) with reactive follicles with no clonal population. Immunohistochemistry confirmed reactive lymph-nodes with polytypic plasma cells, HHV-8 negativity, and no evidence of clonal B-cell or plasma-cell neoplasm.

The overall picture was that of a reactive lymph-node with a prominent interfollicular polyclonal plasma-cell proliferation. Extensive work-up indicated no underlying autoimmune or infectious disease, leading to a diagnosis of plasma-cell variant of Castleman disease.

A repeat-CT at 12-months from symptom onset demonstrated persistent and enlarging lymphadenopathy. PET-CT at 20-months from symptom onset revealed widespread hypermetabolic lymphadenopathy above and below the diaphragm.

The patient commenced systemic corticosteroids (prednisone 40 mg, tapering to 20 mg) and additional urate-lowering therapy (benzbromarone), with partial symptomatic improvement. Hematology initiated siltuximab 11 mg/kg every 3 weeks, with marked clinical and biochemical response: CRP normalized to 10 mg/L, hemoglobin improved from a nadir of 90 g/L to 123 g/L, and total serum globulin fell from >70 g/L to 46 g/L. The patient reported feeling significantly better after three doses. At the most recent review, constitutional symptoms persisted while cutaneous lesions showed gradual improvement. Long-term follow-up is planned under both rheumatology and hematology.

## Discussion

Excess uric acid is a recognized sequela of hematological malignancies, arising through the high cellular turnover rate inherent to leukemia, lymphoma, and plasma-cell dyscrasias. Nucleic acid breakdown from rapid cell turnover or cytolysis leads to increased purine catabolism, generating uric acid as the terminal product; the same mechanism underlying tumor lysis syndrome.^4^ In iMCD, chronic immune activation and polyclonal lymphoproliferation may contribute to a similar hyperuricemia milieu, though this relationship remains incompletely characterized; elevated uric acid in iMCD may alternatively reflect secondary renal impairment.^5^ Given the normal LDH and stable CKD, neither mechanism is entirely satisfactory in this case.

Cutaneous gout most often presents as superficial tophi; involvement of the panniculus is uncommon. Monosodium-urate crystals may deposit in dermal and subcutaneous tissue, but gouty panniculitis is rare, in part because urate is a weak organic acid with difficulty crystallizing in adipose tissue.^6^ A further diagnostic pitfall is that routine formalin fixation dissolves urate crystals, leaving behind nonspecific pale amorphous areas; alcohol fixation is required to preserve the characteristic needle-shaped, negatively birefringent crystals on polarized microscopy.^2^^,^^6^^,^^7^ This necessitates pre-formed clinical suspicion before biopsy to ensure appropriate specimen handling.

The “Polynesian-variant” of iMCD, characterized by HHV-8 negativity, polyclonal plasmacytosis, hypergammaglobulinemia, and systemic inflammation in patients of Pacific heritage has been increasingly recognized, and responsiveness to IL-6 blockade has been described.^1^^,^^8^

This case highlights several diagnostic challenges. Coexisting tophaceous gout initially obscured recognition of an underlying lymphoproliferative disorder, with the cutaneous lesions attributed to advanced gout alone. The severity of panniculitic nodules, significant ongoing systemic symptoms and partial response to prednisone prompted further workup; iMCD is a diagnosis-of-exclusion carrying significant diagnostic delay.

Gouty panniculitis could potentially be associated with an underlying lymphoproliferative disease. The exact mechanism remains poorly elucidated. iMCD should be considered in Pacific patients with unexplained systemic inflammation, lymphadenopathy, and plasma-cell-rich reactive histology. Early multidisciplinary review, integrating dermatology, pathology, rheumatology and hematology can expedite diagnosis and facilitate timely access to targeted therapy.

## Conflicts of interest

None disclosed.
